# A Survey over the Dentists’ and Endodntists’ Approaches towards the Management of Endodontic Emergencies in Mashhad, Iran

**DOI:** 10.7508/iej.2015.04.010

**Published:** 2015

**Authors:** Maryam Bidar, Maryam Gharechahi, Tayebeh Soleimani, Neda Eslami

**Affiliations:** a * Dental Research Center, Dental School, Mashhad University of Medical Sciences, Mashhad, Iran; *; b *Dentist, Mashhad, Iran*

**Keywords:** Emergency, Endodontics, Endodontists, Flare Up, General Dentists, Level of Knowledge

## Abstract

**Introduction:**

Pain or swelling caused by various stages of inflammation/infection of the pulp/periradicular area is among endodontic emergencies. Determining the most effective method of emergency treatment is a challenging issue in endodontics. The goal of this study was to determine and compare the level of knowledge in general dentists and endodontists about endodontic emergency treatment plan in Mashhad, Iran in 2012-2013.

**Methods and Materials:**

In this cross-sectional descriptive study, 152 questionnaires were distributed among 120 general dentists and 32 endodontists of Mashhad. The questionnaire contained two separate parts. The first part included demographic information and in the second part different treatment protocols were suggested for 12 various conditions of pulp/periapical emergencies, and the participants were asked to choose the correct option(s). To determine the relationship between qualitative variables, the chi-square analysis was used. The level of significance was set at 0.05.

**Results:**

There were significant differences between treatment plans presented by general dentists and endodontists about endodontic emergencies, especially in cases of necrotic pulp and subsequent swelling.

**Conclusion:**

Level of knowledge of dentists about the indications of incision and drainage, intra-canal medicament, root filing beyond the apical foramen and antibiotic prescription was not enough. These findings highlight the importance of refreshing courses for general dentists to improve their competency in the management of endodontic emergencies.

## Introduction

Endodontic emergencies include one thirds of dental emergency cases and various strategies are chosen to opposite these conditions including full pulpotomy and extirpation of the pulp in the largest canal (if time permits, cleaning and shaping of all canals or root canal treatment is indicated), intra-canal medication, and prescription of systematic medicines [1]. Treatment of endodontic emergencies is one of the most challenging aspects of clinical dentistry [2]. When a patient has pain or swelling or both caused by various stages of inflammation or infection of the dental pulp or periapical tissues before, during or after root canal treatment (RCT), the situation is considered as an emergency [3]. Since these patients do not usually have scheduled appointments, eliminating the patient’s signs/symptoms and postponing the permanent treatment is the best strategy. This task is an important role of dentists [4, 5]. 

Although there is a great deal of controversy about treatment modalities of endodontic emergencies, little research is available in this issue. Therefore, for dentists it is vital to have enough knowledge and information about the suitable treatment protocols to manage these situations properly.

Currently, educators are interested in planning educational activities based on the identified needs of the target audience. In fact, assessing the learning protocol and its additional requirements is a fundamental step in ensuring the relevance of educational activities to a target audience. This will help the educators in curriculum planning to achieve learning goals and to bring about efficient physicians. In this study, we attempted to determine the learning needs of dentists regarding the management of endodontic emergencies [6].

The aim of this study was to compare the level of knowledge in dentists and endodontists in Mashhad, about treatment of endodontic emergencies.

## Materials and Methods

In this cross-sectional descriptive study (Grant No. 900363) a questionnaire similar to those used in Dorn’s study [3] was applied and updated. Validity of the translated questionnaire was approved by a committee of endodontists and general dentists. Also the reliability of questions was approved. The best method for assessment of the reliability of the questionnaires was test-retest method. The questionnaires were distributed among 12 volunteers (6 dentists and 6 endodontists). After 2 weeks, the questionnaires were redistributed among the same individuals. The Kappa index was calculated to be 0.8. 

All endodontists working in Mashhad (private or academic) participated in this study. Also, general dentists in 12 different regions of Mashhad (10 dentists from each region) were selected by cluster sampling to complete the questionnaires. Totally, 152 questionnaires were distributed among general dentists and endodontists of Mashhad in 2012-2013.

The questionnaire contained two separate parts. The first part included the demographic information of the participants such as age, gender, clinical experience and in the second part different treatment protocols were suggested for 12 various conditions of pulp/periapical emergencies, and the participants were asked to choose the correct option(s). The participants were given an explanation regarding the objective and potential benefit of the study and also they were assured of the confidentiality of their personal information. Data was collected from members of Mashhad Endodontic Association, staffs of Endodontic Department of Mashhad Dental School and general dentists. To determine the relationship between qualitative variables, the chi-square analysis was used. The significance level was set at 0.05. The SPSS software (SPSS version 12, SPSS, Chicago, IL, USA) was used for analysis of data.

## Results

In this study, 152 questionnaires were completed and referred to authors from 120 general dentists and 32 endodontists. Most of the respondents had not completed the demographic information, so it was not possible to evaluate the dependent variables. Descriptive statistics of responses are shown in Tables 1 and 2. Comparison of the responses of dentists and endodontists are listed in Table 3. There was a significant difference between the number of endodontists and dentists that responded according to the standard protocols for management of endodontic emergencies. Some of the most important differences were as follows: Occlusal reduction in cases of irreversible pulpitis and acute apical periodontitis (*P*=0.04) and severe pain and swelling after completion of RCT (*P*=0.007). Prescription of analgesics in cases of necrotic pulp with acute apical periodontitis without swelling (*P*=0.005), necrotic pulp with fluctuant swelling and drainage obtained through the canal (*P*=0.003), necrotic pulp with fluctuant swelling and no drainage (*P*=0.003), necrotic pulp with diffused swelling and no drainage (*P*=0.04), intra-canal medicament in cases of necrotic pulp with fluctuant swelling [with (*P*=0.01) or without (*P*=0.02) drainage obtained through the canal] and necrotic pulp with diffused swelling and drainage (*P*=0.04), incision and drainage in cases of necrotic pulp with diffused swelling and drainage (*P*=0.001), necrotic pulp with diffused swelling and no drainage (*P*=0.03), severe inter-appointment pain and swelling (*P*=0.002) and severe pain and swelling after completion of RCT (*P*=0.009).

## Discussion

The aim of this study was to compare the level of knowledge of dentists and endodontists in Mashhad, Iran in 2012-2013 about their attitude towards the treatment of endodontic emergencies. Although, there is a notable controversy about various treatment modalities of endodontic emergencies, little research is available in this issue. Since there are some differences between treatment plans about endodontic emergencies in different textbooks, determining just one standard treatment procedure is not easy. Standard treatment plans in this study were selected based on the main endodontic textbooks (Pathways of the Pulp, St. Louis: Mosby Co, 2011 [5], and Endodontics: Principles and Practice, 2008 [7]). 

Based on the results of present study, dentists would perform pulpotomy (37.5%) and complete instrumentation (37.5%) in case of irreversible pulpitis without apical periodontitis, while most of the endodontists (84.3%) prefer complete instrumentation. In all references it is obviously indicated that complete instrumentation of root canals is the ideal treatment plan in all endodontic emergency conditions, provided that there is enough time. However, when there is limited time available, partial pulpectomy of anterior teeth and pulpotomy of the largest canal in posterior teeth is recommended, especially when there is no periapical involvement. Pulpotomy of molar teeth is also suggested in case of extremely limited time situations [5, 7, 8]. 

About 13.3% of dentists and 12.5% of endodontists chose pulpotomy in case of irreversible pulpitis with apical periodontitis. Most of the respondents seemed to choose complete instrumentation (60.8% of dentists and 100% of endodontists). In the standard protocol, occlusal reduction is introduced as the standard treatment option, which was selected by endodontists (62.5%) significantly more than general dentists (25.8%) (*P*=0.044).In the study by Dorn *et al.* [3], 80% of the respondents suggested occlusal reduction when there was irreversible pulpitis accompanied by apical periodontitis.

**Table 1:** Descriptive statistics of general dentists (Total=120) and their responses to questioner; N (%)

** Treatment** **Type of ** **Emergency **	**Pulpotomy**	**Partial pulpectomy**	**Complete instrumentation**	**Retreatment**	**Apical surgery **	**Instrument beyond the apex**	**Occlusion reduction**	**Leave tooth open**	**Trephination**	**Incision and drainage**	**Antibiotic**	**Analgesic**	**Intra-canal medicament**
**Irreversible pulpitis-normal periapex**	45 (37.5)	9 (7.5)	45 (37.5)	0 (0)	0 (0)	0 (0)	9 (7.5)	0 (0)	0 (0)	0 (0)	5 (4.1)	33 (27.5)	5 (4.1)
**Irreversible pulpitis-acute apical periodontitis**	16 (13.3)	7 (5.8)	73 (60.8)	0 (0)	0 (0)	2 (1.6)	31 (25.8)	0 (0)	0 (0)	2 (1.6)	21 (17.5)	45 (37.5)	7 (5.8)
**Necrotic pulp with acute apical periodontitis; no swelling **	2 (1.6)	9 (7.5)	80 (66.6)	0 (0)	0 (0)	7 (5.8)	23 (19.1)	2 (1.6)	0 (0)	0 (0)	35 (29.1)	26 (21.6)	31 (25.8)
**Necrotic pulp with fluctuant swelling and drainage obtained through canal**	5 (4.1)	2 (1.6)	59 (49.1)	0 (0)	0 (0)	31 (25.8)	7 (5.8)	33(27.5)	0 (0)	14 (11.6)	40 (33.3)	23 (19.1)	31 (25.8)
**Necrotic pulp with fluctuant swelling and no drainage obtained through canal**	2 (1.6)	2 (1.6)	61 (50.8)	0 (0)	0 (0)	21 (17.5)	5 (4.1)	2 (1.6)	7 (5.8)	47(39.1)	49 (40.8)	23 (19.1)	35 (29.1)
**Necrotic pulp with diffuse swelling and drainage obtained through canal**	2 (1.6)	7 (5.8)	57 (47.5)	0 (0)	0 (0)	16 (13.3)	9 (7.5)	24 (20)	0 (0)	9(7.5)	67 (55.8)	38 (31.6)	33 (27.5)
**Necrotic pulp with diffuse swelling and no drainage obtained through canal**	2 (1.6)	2 (1.6)	49 (40.8)	0(0)	0(0)	21 (17.5)	2 (1.6)	14(11.6)	12(10)	28(23.3)	54 (45)	33 (27.5)	35 (29.1)
**Sever inter-appointment pain **	0 (0)	0 (0)	45 (37.5)	0(0)	0(0)	2 (1.6)	57 (47.5)	5 (4.1)	2 (1.6)	0 (0)	19 (15.8)	66 (55)	31 (25.8)
**Sever inter-appointment pain and swelling **	0 (0)	9 (7.5)	49 (40.8)	0(0)	2(1.6)	0 (0)	40 (33.3)	2 (1.6)	0 (0)	7 (5.8)	57 (47.5)	71 (59.1)	40 (33.3)
**Sever pain after completion of RCT**	0 (0)	0 (0)	2 (1.6)	7(5.8)	0(0)	0 (0)	38 (31.6)	2 (1.6)	0 (0)	0 (0)	19 (15.8)	96 (80)	7 (5.8)
**Sever pain and swelling after completion of RCT**	0 (0)	0 (0)	2 (1.6)	31(25.8)	2(1.6)	0 (0)	19 (15.8)	0 (0)	0 (0)	7 (5.8)	73 (60.8)	75 (62.5)	5 (4.1)
**Pain in failure of RCT**	0 (0)	0 (0)	5 (4.1)	89(74.1)	45(37.5)	0 (0)	2 (1.6)	0 (0)	0 (0)	0 (0)	19 (15.8)	31 (25.8)	2 (1.6)

**Table 2:** Descriptive statistics of endodontists (Total=32) and their responses to questioner; N (%)

** Treatment** **Type of ** **Emergency **	**Pulpotomy**	**Partial pulpectomy**	**Complete instrumentation**	**Retreatment**	**Apical surgery **	**Instrument beyond the apex**	**Occlusion reduction**	**Leave tooth open**	**Trephination**	**Incision and drainage**	**Antibiotic**	**Analgesic**	**Intra-canal medicament**
**Irreversible pulpitis-normal periapex**	15 (46.8)	4 (12.5)	27 (84.3)	0 (0)	0 (0)	0 (0)	3 (9.3)	0 (0)	1 (3.1)	0 (0)	0 (0)	17 (53.1)	3 (9.3)
**Irreversible pulpitis-acute apical periodontitis**	4 (12.5)	4 (12.5)	32 (100)	0 (0)	0 (0)	0 (0)	20 (62.5)	0 (0)	0 (0)	0 (0)	1 (3.1)	24 (75)	11 (34.3)
**Necrotic pulp with acute apical periodontitis; no swelling **	0 (0)	1 (3.1)	32 (100)	0 (0)	0(0)	0 (0)	16 (50)	0 (0)	0 (0)	0 (0)	5 (15.6)	24 (75)	17 (53.1)
**Necrotic pulp with fluctuant swelling and drainage obtained through canal**	0 (0)	0 (0)	27 (84.3)	0 (0)	0 (0)	3 (9.3)	13 (40.6)	1 (4.2)	1 (4.2)	11 (34.3)	9 (28.1)	24 (75)	24 (75)
**Necrotic pulp with fluctuant swelling and no drainage obtained through canal**	0 (0)	0 (0)	28 (87.5)	0 (0)	0 (0)	5 (15.6)	12 (37.5)	0 (0)	1 (3.1)	25 (78.1)	15 (46.8)	24 (75)	24 (75)
**Necrotic pulp with diffuse swelling and drainage obtained through canal**	0 (0)	0 (0)	28 (87.5)	0 (0)	0 (0)	1 (3.1)	11 (34.3)	1 (3.1)	0 (0)	16 (50)	29 (90.6)	24(75)	21(65.6)
**Necrotic pulp with diffuse swelling and no drainage obtained through canal**	0 (0)	0 (0)	28 (87.5)	0 (0)	0 (0)	8 (25)	13 (40.6)	0 (0)	3 (9.3)	20 (62.5)	24 (75)	21 (65.6)	20 (62.5)
**Sever inter-appointment pain **	0 (0)	0 (0)	24 (75)	0 (0)	0 (0)	1 (3.1)	20 (62.5)	1 (3.1)	0 (0)	0 (0)	9 (28.1)	24 (75)	15 (46.8)
**Sever inter-appointment pain and swelling **	0 (0)	1 (3.1)	25 (78.1)	0 (0)	0 (0)	1 (3.1)	16 (50)	3 (9.3)	0 (0)	13 (40.6)	25 (78.1)	31 (96.8)	23 (71.8)
**Sever pain after completion of RCT**	0 (0)	0 (0)	0 (0)	0 (0)	0 (0)	0 (0)	17 (53.1)	1 (3.1)	0 (0)	0 (0)	5 (15.6)	32 (100)	0 (0)
**Sever pain and swelling after completion of RCT**	0 (0)	0 (0)	0 (0)	0 (0)	0 (0)	0 (0)	19 (59.3)	1 (1.3)	3 (9.3)	11 (34.3)	32 (100)	31 (96.8)	0 (0)
**Pain in failure of RCT**	0 (0)	0 (0)	0 (0)	32 (100)	11 (34.3)	1 (3.1)	7 (21.8)	0 (0)	1 (3.1)	1 (3.1)	8 (25)	15 (46.8)	8 (25)

**Table 3:** Comparison of the responses of general dentists (Total=120) and endodontists (Total=32)

**Emergency condition**	**Standard protocol**	**Endodontist N (%)**	**General dentist N (%)**	***P*** **-value**
**Irreversible pulpitis-normal periapex**	Partial pulpectomy Analgesic	4 (12.5) 17 (53.1)	9 (7.5) 33 (27.5)	0.5588 0.1345
**Irreversible pulpitis-acute apical periodontitis**	Complete instrumentation Occlusion adjustment Analgesic	32 (100) 20 (62.5) 24 (75)	73 (60.8) 31 (25.8) 45(37.5)	0.1744 0.0446 0.0869
**Necrotic pulp with acute apical periodontitis-no swelling**	Complete instrumentation Analgesic Intracanal medicament	32 (100) 24 (75) 17 (53.1)	80 (66.6) 26 (21.6) 31 (25.8)	0.2640 0.0051 0.1007
**Necrotic pulp with fluctuant swelling and drainage obtained through the canal**	Complete instrumentation Incision and drainage Analgesic Intracanal medicament	27 (84.3) 11 (34.3) 24 (75) 24 (75)	59 (49.1) 14 (11.6) 23 (19.1) 31 (25.8)	0.1708 0.0719 0.0030 0.0126
**Necrotic pulp with fluctuant swelling and no drainage obtained through the canal**	Complete instrumentation Instrument past the apex Incision and drainage Analgesic Intracanal medicament	28 (87.5) 5 (15.6) 25 (78.1) 24 (75) 24 (75)	61 (50.8) 21 (17.5) 47 (39.1) 23 (19.1) 35 (29.1)	0.1577 0.9299 0.0806 0.0030 0.0268
**Necrotic pulp with diffuse swelling and drainage obtained through canal**	Complete instrumentation Incision and drainage Antibiotic Analgesic Intracanal medicament	28 (87.5) 16 (50) 29 (90.6) 24 (75) 21 (65.6)	57 (47.5) 9 (7.5) 29 (55.8) 38 (31.6) 33 (27.5)	0.1081 0.0014 0.2025 0.374 0.042
**Necrotic Pulp with diffuse Swelling and no drainage obtained through canal**	Complete instrumentation Instrument past the apex Incision and drainage Antibiotic Analgesic Intracanal medicament	28 (87.5) 8 (25) 20 (62.5) 24 (75) 21 (65.6) 20 (62.5)	49 (40.8) 21 (17.5) 28 (23.3) 54 (45) 33 (27.5) 35 (29.1)	0.0549 0.5485 0.0308 0.2023 0.0420 0.0846
**Sever pain in inter appointment **	Complete instrumentation Occlusion reduction Analgesic	24 (75) 20 (62.5) 24 (75)	45 (37.5) 57 (47.5) 66 (55)	0.0869 0.4903 0.4238
**Sever pain and swelling in inter appointment**	Complete instrumentation Instrument past the apex Incision and drainage Analgesic Intracanal medicament	25 (78.1) 1 (3.1) 13 (40.6) 31 (96.8) 23 (71.8)	49 (40.8) 0 (0) 7 (5.8) 71 (59.1) 40 (33.3)	0.1018 0.1505 0.0021 0.1877 0.0723
**Sever pain** ** after completion of RCT**	Occlusion adjustment Analgesic	17 (53.1) 32 (100)	38 (31.6) 96 (80)	0.2205 0.5405
**Sever pain and swelling after completion of RCT**	Occlusion adjustment Incision and drainage Analgesic	19 (59.3) 11 (34.3) 31 (96.8)	19 (15.8) 7 (5.8) 75 (62.5)	0.0075 0.0091 0.2497
**Pain in failure of RCT**	Analgesic Retreatment or apical surgery	15 (46.8) 32 (100)	31 (25.8) 120 (100)	0.2137 0.2428

In necrotic teeth without swelling, the most common and standard emergency treatment includes complete instrumentation, occlusal reduction, prescription of analgesics and application of intra-canal medicament [3, 5]. Treatment preference of endodontists in this condition was completely in accordance with this standard treatment (100% of cases); however, there was no significant difference between the two groups. 

The standard emergency treatment of teeth with necrotic pulp, fluctuant swelling and drainage through the canal includes complete instrumentation [5, 7, 9]. Most of the endodontists (84.3%) and nearly half of the dentists chose this protocol. In dentists group, more number of respondents (25.8%) chose the "filing beyond the apex" while it was chosen by 9.3% of endodontists. Filing beyond the radiographic apex is more recommended when no drainage can be obtained through the canal [7]. Several authors have recommended confining the instruments to canal limits in cases of necrotic pulp with drainage through the canal to prevent the pushing of necrotic debris into the periapical region and thus further exacerbation of an already acute situation [10]. Almost 27.5% of dentists preferred to "Leave tooth open" when there is pulpal necrosis and swelling. However, none of the endodontists chose this treatment option. Since the opened canal is a suitable place for aggregation of bacteria, food debris and viruses, this treatment modality is not considered as a standard option [11,12]. Selden and Parris [13] observed that leaving tooth open would result in 11% more secondary periapical involvement. It is recommended to leave the teeth with active drainage open with rubber dam for a short time before sealing them to prevent later flare-up [7]. Weine *et al.* [14] found that contemporary restoration of teeth with vital pulp in the emergency visit would result in less exacerbation and fewer treatment visits [14]. Another standard treatment option in this endodontic emergency includes incision and drainage, which was selected by only 11.6% of dentists and 34.3% of endodontists. Sometimes more than one abscess is possible. One is connected to apex and the other one is separately in the vestibule. Since they are not connected with each other, drainage through the tooth is not enough and incision is also necessary [7]. Therefore the respondents should have chosen incision and drainage option as a classic answer; even in cases when swelling is not considerable, it is better to do incision to reduce pain and swelling [9].

Managing the emergency condition when there is pulpal necrosis with fluctuant swelling without drainage through the canal, includes complete canal instrumentation, filing beyond the apex with a medium sized file, incisions and drainage, placement of intra-canal medicament and prescribing analgesics according to the standard protocols [5, 7]. Most of the endodontists (more than 80%) followed the standard protocol, but dentist’s awareness of standard emergency treatment methods in the same condition was lower. Both groups similarly preferred antibiotic prescription and filing beyond the apex. It is necessary to remind that there is no need to prescribe antibiotic in this emergency condition; however, results of our study indicate that nearly 50% of each group did not follow the correct protocol for antibiotic prescription.

In necrotic teeth with diffused swelling without drainage through canal, most of respondents preferred complete instrumentation with filing beyond the apex (40.8% of dentists and 87.5% of endodontists) which is in accordance with the standard treatment plan. One of the other standard treatment options in these cases is incision and drainage that was chosen by 62.5% of endodontists. If there is fluctuant swelling, this number rises up to 78.1%. This difference can be due to the worldwide agreement about incising the fluctuant swelling to obtain drainage. However, there is still controversy regarding incision of diffused swellings [7]. It is necessary to prescribe antibiotics in cases with diffuse swelling. In this study 83% of endodontists and 50% of dentists recommended antibiotics in this condition. Although, higher percentage of endodontists prescribed antibiotics in necessary cases, unnecessary prescription of antibiotics by endodontists was still high. In fact, more than 57% of endodontists recommended antibiotics in cases without true indication. Similarly, Navabizadeh *et al.* [10], reported that only 29% of dentists had adequate knowledge about the correct protocol for antibiotic prescription. Kakoei *et al.* [11] showed that high percentage of responders prescribe antibiotic for fever and diffused swelling. However, some situations such as acute pulpitis, chronic periapical lesions and marginal gingivitis were irrationally prescribed.

Another significant finding in this study was that endodontists used calcium hydroxide as an intra-canal medicament significantly more than dentists in all different conditions of necrotic teeth. Endodontists mostly used intra-canal medicaments in cases with pulp necrosis and fluctuant swelling (75%), while dentists used them when there was severe pain and swelling between appointments (33.3%).

In cases of severe inter-appointment pain, standard treatment included re-instrumentation, occlusal reduction and analgesic prescription [5, 7]. Most of the endodontists (75%) and 37.5% of dentists recommended re-instrumentation in this condition. Also occlusal reduction and analgesic prescription were recommended by most of the respondents especially endodontists, which was consistent with standard treatment. Usually, when there is inter-appointment pain, the temporary filling should be checked for traumatic occlusal contacts [12]. 

In cases of flare-ups with severe pain and swelling, the standard treatment protocol includes re-instrumentation, filing beyond the apex, incision and drainage, analgesic prescription and placement of intracranial medicament [5, 7]. More than 75% of endodontists and less than 50% of dentists chose re-instrumentation in these cases. The teeth with already necrotic pulps and swelling between appointments and also those with swelling after completion of RCT, could be managed by incision and drainage [7]. In the present study, only 5.8% of dentists and 40.6% of endodontists recommended incision and drainage in these conditions, and the difference between the two groups was statistically significant (*P*=0.0021).

In cases with severe pain and swelling after RCT, 5.9% of general dentists and 33.3% of endodontists chose incision and drainage and the difference was also significant (*P*=0.009).

Retreatment is required in cases with unsuccessful previous RCT. When there is an acute apical abscess with an unsuitable RCT and no coronal access for retreatment, apical surgery is required [7, 13, 14]. These two treatment options were recommended by 100% of all respondents in cases of failed RCT. It should be noted that pain after RCT is not considered as a failure of treatment and is regarded as post-operative flare-up. 

It is important to note that antibiotic prescription was a very common treatment plan for inter-appointment pain or at the completion of RCT, and was suggested by 47% of dentists and more than 78% of endodontists. However, this was not consistent with the standard treatment plans in this study [5, 7]. Also a significant number of dentists would leave the teeth open in cases of necrosis with drainage through canal or diffused swelling with no drainage, which was not according to standards.

One limitation of this study was the cross-sectional design. Also the anterior and posterior teeth were not separately evaluated. On the other hand, it was not possible to study the dependent variables, because most of the respondents had not completed the demographic information.

## Conclusion

The results of this study revealed that there are significant differences between treatment plans offered by dentists and endodontists in cases of endodontic emergencies. Level of knowledge of dentists about indications of incision and drainage, intra-canal medicament and antibiotic prescription was not enough. This emphasizes the importance of refreshing courses for general dentists to improve their competency in the management of endodontic emergencies.
